# Calcium Increase and Substance P Release Induced by the Neurotoxin Brevetoxin-1 in Sensory Neurons: Involvement of PAR2 Activation through Both Cathepsin S and Canonical Signaling

**DOI:** 10.3390/cells9122704

**Published:** 2020-12-17

**Authors:** Ophélie Pierre, Maxime Fouchard, Paul Buscaglia, Nelig Le Goux, Raphaël Leschiera, Olivier Mignen, Joachim W. Fluhr, Laurent Misery, Raphaële Le Garrec

**Affiliations:** 1 EA4685 Laboratory of Interactions Neurons-Keratinocytes (LIEN), Faculty of Medicine and Health Sciences, University Brest, F-29200 Brest, France; maxime.fouchard@gmail.com (M.F.); raphael.leschiera@univ-brest.fr (R.L.); Joachim.Fluhr@charite.de (J.W.F.); laurent.misery@chu-brest.fr (L.M.); rlegarrec@univ-brest.fr (R.L.G.); 2 Department of Dermatology, University Hospital of Brest, F-29200 Brest, France; 3 InsermUMR1227, Lymphocytes B et Autoimmunity, University Brest, F-29200 Brest, France; paul.buscaglia@gmail.com (P.B.); Nelig.LeGoux@univ-brest.fr (N.L.G.); olivier.mignen@univ-brest.fr (O.M.); 4 Department of Dermatology and Allergology, Universitaetsmedizin Charit Berlin, D-10117 Berlin, Germany

**Keywords:** brevetoxin, ciguatoxin, sensory disorders, substance P, cathepsin S, PAR2, biased pathway, canonical pathway

## Abstract

Red tides involving *Karenia brevis* expose humans to brevetoxins (PbTxs). Oral exposition triggers neurotoxic shellfish poisoning, whereas inhalation induces a respiratory syndrome and sensory disturbances. No curative treatment is available and the pathophysiology is not fully elucidated. Protease-activated receptor 2 (PAR2), cathepsin S (Cat-S) and substance P (SP) release are crucial mediators of the sensory effects of ciguatoxins (CTXs) which are PbTx analogs. This work explored the role of PAR2 and Cat-S in PbTx-1-induced sensory effects and deciphered the signaling pathway involved. We performed calcium imaging, PAR2 immunolocalization and SP release experiments in monocultured sensory neurons or co-cultured with keratinocytes treated with PbTx-1 or P-CTX-2. We demonstrated that PbTx-1-induced calcium increase and SP release involved Cat-S, PAR2 and transient receptor potential vanilloid 4 (TRPV4). The PbTx-1-induced signaling pathway included protein kinase A (PKA) and TRPV4, which are compatible with the PAR2 biased signaling induced by Cat-S. Internalization of PAR2 and protein kinase C (PKC), inositol triphosphate receptor and TRPV4 activation evoked by PbTx-1 are compatible with the PAR2 canonical signaling. Our results suggest that PbTx-1-induced sensory disturbances involve the PAR2-TRPV4 pathway. We identified PAR2, Cat-S, PKA, and PKC that are involved in TRPV4 sensitization induced by PbTx-1 in sensory neurons.

## 1. Introduction

Brevetoxins (PbTxs) are a family of cyclic and lipophilic polyether compounds. These phytoplanktonic neurotoxins are synthetized during algal blooms, especially during red tides involving *Karenia brevis*. PbTxs are accumulated in molluscs (e.g., oysters, mussels or clams) and could be aerosolized. The main routes of human exposure are the oral route, resulting in neurotoxic shellfish poisoning (NSP), and the inhaled route, resulting in a respiratory syndrome [1]. The largest outbreaks of NSP happened in the USA (North Carolina and Gulf of Mexico, especially Florida) and in New Zealand following red tides (algal blooming) [1]. NSP syndrome includes gastrointestinal symptoms and neurological signs such as abdominal pain, paresthesia, cold allodynia, vertigo, ataxia, muscle pain, weakness and headache [1,2]. Those symptoms occur within few minutes to several hours after seafood ingestion and last 1 to 3 days [2]. Currently, the NSP incidence is low due to prevention, information and the monitoring of algal blooming [3]. In contrast, airway irritation following respiratory PbTx-exposure increase periodically including emergency room admissions [4]. Inhalation of PbTx aerosols result in respiratory symptoms especially in persons with underlying airway diseases such as asthma. Upper-airway symptoms include cough, sneezing and rhinorrhea, involving sensory fiber activation. Lower-airway symptoms include chest tightness, wheezing and shortness of breath [5,6,7,8]. Burning sensations in nose and throat, associated with itch on the facial skin, watery eyes and neurological symptoms such as headache, have been reported [9]. These symptoms support the hypothesis that PbTx exert sensory effects through inhalation. Current treatment is only symptomatic due to the lack of a specific antidote for PbTx intoxications. Climatic changes and global warming may increase the occurrence of algal blooming of *K. brevis* [10] and expand the affected areas in Europe [11]. Beyond effects on humans, PbTxs have destructive effects on the fauna including turtles [12], marine mammals [13,14,15], fishes and birds.

Currently, several PbTx derivatives have been identified [3], including PbTx-1 which is the most potent congener whereas PbTx-2 and PbTx-3 are the most abundant in aerosols [16,17]. Mechanistically, PbTxs interact with site 5 of the alpha subunit of the voltage-dependent sodium channels (Na_v_) including tetrodotoxin (TTX)-sensitive Na_v_ channels (TTX-s Na_v_) and the TTX-resistant Na_v_ channels (TTX-r Na_v_) [18,19,20,21], which are largely expressed in sensory nerves [22]. PbTx binding causes conformational changes of the channel, shift in the activation voltage and impairment of inactivation. This, in turn, increases sodium entrance, leading to membrane cell hyperexcitability and subsequently to sensory disturbances [23]. Structural and functional analogs of PbTxs, called ciguatoxins (CTXs), compete with PbTxs for site 5 and cause similar modifications of channel gating [21,23]. They induce a syndrome called ciguatera fish poisoning (CFP), similar to NSP initially described by McFarren et al. [24]. In a previous study, our team showed that Pacific-ciguatoxin-2 (P-CTX-2) is able to induce the release of neuropeptides including substance P (SP) and calcitonin gene-related peptide (CGRP) in a co-culture model of human keratinocytes and rat sensory neurons [25]. Those neuropeptides and particularly SP play a major role in pruritus [26], nociception, pain [27] and lead to neurogenic inflammation [26,28]. A second study demonstrated that P-CTX-2-induced SP release and increase of intracellular calcium concentration ([Ca^2+^]_i_) in sensory neurons. Those effects are mediated by the increase of the protease cathepsin S (Cat-S) activity and the activation of the protease-activated receptor 2 (PAR2) in a Na_v_-dependent manner [29]. The involvement of these mediators in the pathophysiology of PbTx-induced sensory effects has never been studied.

PAR2 belongs to a family of four cell surface receptors coupled to G protein (PAR1 to PAR4). This receptor is highly expressed in the membrane of sensory neurons (around 60%) particularly in neurons of small and medium diameter [26], as well as in keratinocytes [30]. PAR2 activation can mediate pruritus [28] and pain [31,32]. PAR2 is activated by several endogenous proteases including Cat-S mainly released from macrophages/microglia [33,34], elastase produced from neutrophils [35], trypsin from pancreas [36] and tryptase from mast cells [30]. PAR2 activation in primary sensory neurons triggers neuropeptide release, such as SP and CGRP, leading to neurogenic inflammation in the peripheral nervous system [26]. In total, 40% of dorsal root ganglion (DRG) neurons co-express CGRP and PAR2 whereas 30% of DRG neurons co-express SP and PAR2 [26]. PAR2 couples to multiple G-protein subtypes including Gαq, Gαs, and Gα12/13, promoting diverse signaling pathways. The two main modes of activation and subsequent signaling pathways are described, namely canonical [37] and biased activation [38,39].

The canonical activation of PAR2 is mediated by proteases including trypsin, a full-agonist. Trypsin cleaves PAR2 at a specific site to reveal a new N-terminal part and a tethered ligand Ser-Leu-Ile-Gly-Lys-Val (SLIGKV) in human or Ser-Leu-Ile-Gly-Arg-Leu (SLIGRL) in rat and mouse, which is able to initiate canonical signaling [40]. On the one hand, activated PAR2 couples to Gαq/11 and activates phospholipase C (PLC). PLC hydrolyses phosphatidyl inositol-bisphosphate (PIP2) which release inositol-1,4,5-triphosphate (IP3) and diacylglycerol (DAG) from the membrane. IP3 acts on its receptor named inositol triphosphate receptor (IP3R) which leads to a prompt Ca^2+^ mobilization from IP3R-dependent endoplasmic reticulum stores, and activation of protein kinases A, C and D (PKA, PKC and PKD) [41,42,43,44]. This leads to the sensitization of the transient receptor potential channels (TRP) including transient receptor potential vanilloid 1 (TRPV1) [42,45,46], transient receptor potential ankyrin 1 (TRPA1) [47] and transient receptor potential vanilloid 4 (TRPV4) [48] in sensory nerve fibers contributing to sensory disorders. On the other hand, full agonists signal through β-arrestin, which is associated with PAR2 internalization, and phosphorylation of extracellular signal-regulated kinases 1 and 2 (ERK1/2) [49,50,51,52]. The signalosome complex prevents ERK translocation in the nucleus and allows phosphorylation of cytosolic substrates. Next, PAR2 moves into lysosomes where it is probably degraded or recycled. Internalization of PAR2 desensitizes cells to new PAR2 challenges. Re-sensitization occurs through PAR2 re-expression at the plasma membrane by intracellular store mobilization and/or de novo synthesis of the receptor [36]. Several proteases called biased PAR2 agonists including Cat-S or neutrophil elastase cleave PAR2 at distinct sites [34,53] from the canonical trypsin site, leading to a different outcome called biased signaling. Cat-S is a lysosomal enzyme, optimally active at acid and neutral pH. Cat-S regulates the process of maturation for the class II major histocompatibility complex in antigen presenting cells [54,55]. Cat-S elicits itch through PAR2 activation [56]. The Cat-S cleavage leads to the Gαs-dependent formation of cyclic adenosine monophosphate (cAMP) and activation of PKA. This process, in turn, sensitizes TRP channels (e.g., TRPV4 facilitating calcium entry) and contributing to inflammatory pain [53,57]. Biased PAR2 agonists including Cat-S also signal through PKD and Gβγ to induce pain [58]. In contrast to classical PAR2 agonists, they do not signal through the β-arrestin pathway and do not internalize PAR2 [53]. Biased PAR2 agonists do not signal through Gαq and calcium mobilization from internal stores [53,58]. However, in the cell line Hela transfected with PAR2 and human epidermal keratinocytes, Cat-S was able to induce [Ca^2+^]_i_ increase, generate inositol monophosphate (a downstream metabolite of IP3) and activate PKC [34]. In contrast to neutrophil elastase, Cat-S was able to couple PAR2 to Gαq, although at higher concentrations than those required to couple PAR2 to Gαs [58]. These data suggest that the biased PAR2 signaling induced by Cat-S could include some actors of the classical PAR2 signaling. 

Downstream signaling pathways of PAR2 activation by PbTxs are unknown. Since PbTxs are structural and functional analogs of CTXs, PbTx-1 and P-CTX-2 were tested in our study. Our hypotheses were (i) PbTx-1-induces an increase of [Ca^2+^]_i_ and SP release in sensory neurons; (ii) Na_v_, PAR2 and Cat-S are involved in those effects and (iii) Actors of PAR2 downstream pathways (e.g., PKC, IP3R, PKA and TRPV4) are involved downstream of PAR2 activation induced by PbTx-1 in sensory neurons.

We performed single cell cytosolic calcium video imaging and immunocytochemical experiments on DRG sensory neurons whereas experiments of SP release were conducted with an optimized co-culture model of rat DRG neurons and human epidermal keratinocytes. In the skin, neuropeptides are released by free intra-epidermal nerve endings that are in close proximity to keratinocytes [59]. In previous work, we reported that P-CTX-2 is able to increase a SP release from sensory neurons but not from keratinocytes [29]. However, levels of SP released by DRG sensory neurons were inadequate to study the molecular actors. In contrast, levels of P-CTX-2-induced SP released by a co-culture of DRG sensory neurons and keratinocytes were six-fold higher than in DRG sensory neurons alone, becoming sufficient to study the molecular actors. This optimized model allows us to decipher the mechanisms of PbTx-1-induced SP release.

## 2. Materials and Methods

### 2.1. Primary Cell Cultures

All experiments were approved by local authorities and in accordance with the French Ministry of Agriculture and the European Communities Council Directive 2010/63/UE. Human experiments and sample collections had local authorization, referenced under DC 2008-214 at the “Ministere de la Recherche” in France and were conducted following the principles of the Declaration of Helsinki.

Rat sensory neurons were obtained from DRG of neonatal rats as previously described [25]. Briefly, DRG were extracted from newborn Wistar rats between 3 and 5 days after birth, then enzymatically (collagenase IV, 200 units/mL) and mechanically dissociated. DRG suspension was filtered through 100 µm cell strainer before seeding in 96-well plates or on glass coverslips coated with poly-L-lysine (PLL).

Human skin samples were obtained from healthy adult donors undergoing plastic surgery. The donors had given their written after informed consent. Dissociated human epidermal keratinocytes were obtained as previously described [60] with minor modification including the dispase (20 UI/mL) used for enzymatic digestion overnight at 4 °C. Keratinocytes between 2 and 5 passages were maintained in complete Keratinocyte-Serum Free Medium (KSFM) for 24 h and then, differentiated for 14–16 h in mixture of Dulbecco’s Modified Eagle Medium (DMEM) and DMEM plus Ham’s F12 media (DMEM-F12) (DMEM-DMEM/F12) (1:1).

The co-culture model was performed as previously described [25] with minor modifications. Briefly, DRG neurons were seeded at a rate of one newborn rat for 10 or 12 wells in 96-well plates in DMEM/DMEM/F12 (1:1) supplemented with Normocin^®^ (100 µg/mL), B27 (20 µL/mL), nerve growth factor (NGF) (100 ng/mL), insulin (4 µg/mL), brain-derived neurotrophic factor (BDNF) (20 ng/mL) and hydrocortison (10 ng/mL). After 3–5 days of culture, the medium was removed, and 20,000 human non-differentiated keratinocytes per well were seeded in complete KSFM. Co-cultures were maintained for 24 h at 37 °C in a 5% CO_2_ humidified atmosphere to allow keratinocyte attachment and then, the medium was replaced by DMEM: DMEM/F12 (1:1) to induce keratinocyte differentiation for 14–16 h.

### 2.2. Single Cell Cytosolic Calcium Video Imaging

DRG neurons were cultured on glass coverslips coated with PLL and maintained at 37 °C. Then, 1 day after, DRG neurons were placed in a recording buffer (135 mM NaCl, 5 mM KCl, 1 mM MgCl_2_, 1.8 mM CaCl_2_, 10 mM HEPES, 10 mM glucose and pH adjusted at 7.45 with NaOH) and loaded with 4 µM of Fura-2/AM (Molecular Probes, Invitrogen, Cergy Pontoise, France) for 30 min at 37 °C. After washing, ratiometric images of calcium signals (340/380 nm) were obtained with the microscope IX71 Olympus equipped with a monochromator illumination system (Polychrome V, TILL Photonics). Emission was captured through a 415 DCLP dichroic mirror, by a 14-bit CCD camera (EXiBlue, Qimaging). Image acquisition and analysis were performed with the Metafluor 6.3 software (Universal Imaging, West Chester, PA, USA) at room temperature (RT). 

At least 50 to 150 regions of interest were carefully defined with single neuron for each condition of each experiment. Each antagonist (listed in supplementary materials—Table S1) was added 10 min before image acquisition, then PbTx-1 was injected manually and data were recorded for 25 min. Only one challenge per coverslip was made (i.e., one antagonist pretreatment before one PbTx-1 treatment). Background subtraction and calculation of the resulting 340/380 ratio images were performed offline (ΔF/F0) (i.e., with the formula ΔF/F0 = (F − F0)/F0). Ca^2+^ entry was quantified after value normalization (ΔF/F0) with the exception of basal Ca^2+^ concentrations estimated as the average of initial F340 nm/F380 nm values. 

Cells were considered to respond when they exhibited a ΔF/F0 increase of at least 0.15. For the responding cells, ΔF/F0 amplitude values for each phase of the PbTx-1-elicited [Ca^2+^]_i_ increase were reported, using maximum ΔF/F0 for the first and the second calcium transients and the average ΔF/F0 obtained between 20 and 25 min following PbTx-1 application for the long-lasting phase also called plateau. For each phase, the inhibitory effects of antagonists were assessed first, on their ability to decrease percentages of cell responding to PbTx-1 and secondly, the associated amplitude values using normalized data, considering as 100% the average data obtained with PbTx-1 alone (control condition). Normalization to the control condition (PbTx-1 alone) was thus applied experiment by experiment which resulted in a 100% value for the PbTx-1 condition without SEM. The “*n*” represented the number of independent experiments in which 50 to 150 neuronal cells have been integrated to the calcium analysis. 

### 2.3. SP Enzyme Immunoassay (EIA)

Levels of SP in co-culture supernatants were quantified using SP EIA Kit (R&D systems, Minneapolis, MN, USA), according to the manufacturer’s instructions and were expressed in pg/mL. Each experiment was performed in duplicate. The values from the control conditions were subtracted from those obtained under the different PbTx conditions. The average levels of released SP (in pg/mL) obtained under the different antagonist conditions (listed in supplementary materials—Table S1) were normalized to the average data obtained for PbTx-1 alone. The SP measurement data were expressed as mean ± standard error of the mean (SEM) of at least three separate experiments.

### 2.4. Immunocytochemical Quantification of PAR2 Internalization

DRG neurons plated on 96-well plates were maintained in culture for 1 day. Neurons were treated with 10 nM of P-CTX-2 for 20 min with or without pre-treatment of 10 nM of Z-FL-COCHO. Cells were then fixed in paraformaldehyde 2% and were not permeabilized in order to quantify the membrane PAR2 expression. Unspecific sites were blocked in PBS-milk 5%. PAR2 immunolabeling was performed using the mouse monoclonal [SAM11] primary antibody (Ab184673, Abcam, Paris, France, 1:100) and an anti-mouse secondary antibody coupled to horseradish peroxidase (HRP) (Jackson Immuno Research Europe, Suffolk, England). HRP substrate was added and the reaction was allowed to proceed for 20 min. The relative levels of membrane PAR2 expression were obtained by measuring the absorbance levels at 492 nm, which were normalized to the total amount of cells via Janus Green whole-cell staining. Each experiment was performed in triplicate. Data were expressed as mean ± standard error of the mean (SEM) of at least three separate experiments.

### 2.5. Statistical Analysis

The statistical analyses were conducted with GraphPad Prism 6.0 (San Diego, CA, USA). The details are provided in the figure captions. Shapiro–Wilk normality test was performed and in case of non-normal distribution, a non-parametric approach was chosen. The differences were considered statistically significant with *p* < 0.05. 

## 3. Results

### 3.1. PbTx-1-Induces an Increase of [Ca^2+]^_i_ in DRG Sensory Neurons

**Figure 1:**
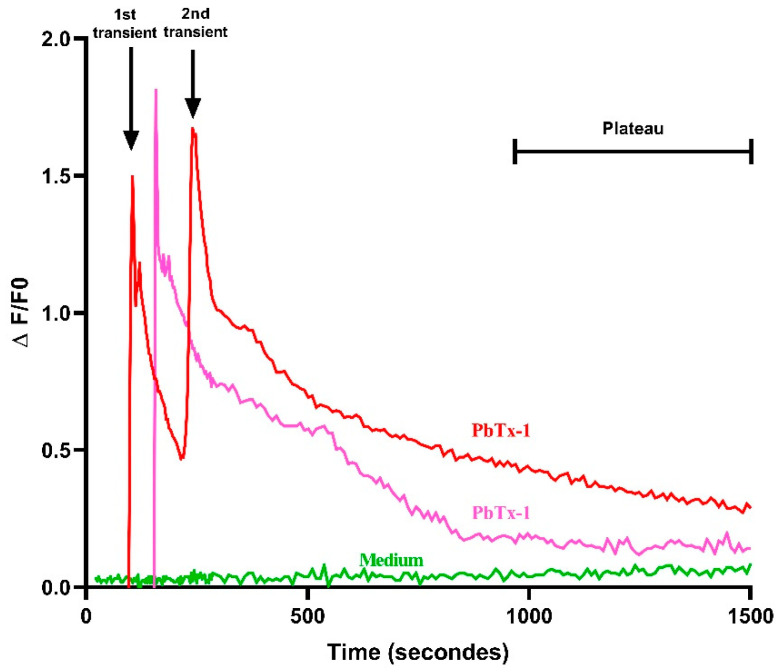
Representative profiles of PbTx-1-induced [Ca^2+^]_i_ increase in DRG sensory neurons highlighting different subpopulations of sensory neurons. Profiles in red and pink represent PbTx-1-induced [Ca^2+^]_i_ increase comprising of three phases (first transient, second transient and plateau) and two phases (transient and plateau), respectively. Medium (control) did not induce [Ca^2+^]_i_ increase as represented in the green line. [Ca^2+^]_i_: intracellular calcium concentration; PbTx-1: brevetoxin-1.

PbTx-1 induced [Ca^2+^]_i_ increase in 54% of DRG neurons. In 60% of responding neurons, the response was a remarkable multiphasic and long-lasting [Ca^2+^]_i_ increase (Figure 1). Immediately post injection of 1 µM of PbTx-1, [Ca^2+^]_i_ strongly increased, reaching an average maximum amplitude of 1.196 ± 0.51 (mean ± SD; *n* = 44). This first transient, called 1st peak, was rapidly down-regulated. The second transient, called 2nd peak, reached progressively an averaged maximum amplitude of 1.256 ± 0.53 (mean ± SD; *n* = 44) and continued in a long-lasting phase named plateau, which achieved an averaged amplitude of 0.492 ± 0.49 (mean ± SD; *n* = 44). In 40% of responding sensory neurons, PbTx-1 induced a monophasic [Ca^2+^]_i_ increase finishing by a long-lasting phase (also called plateau). The vehicle control (medium) did not induce a calcium response.

### 3.2. PbTx-1-Induced [Ca^2+^]_i_ Increase and SP Release in DRG Neurons Are Mediated by Na_v_ Channels, Cat-S and PAR2

**Figure 2:**
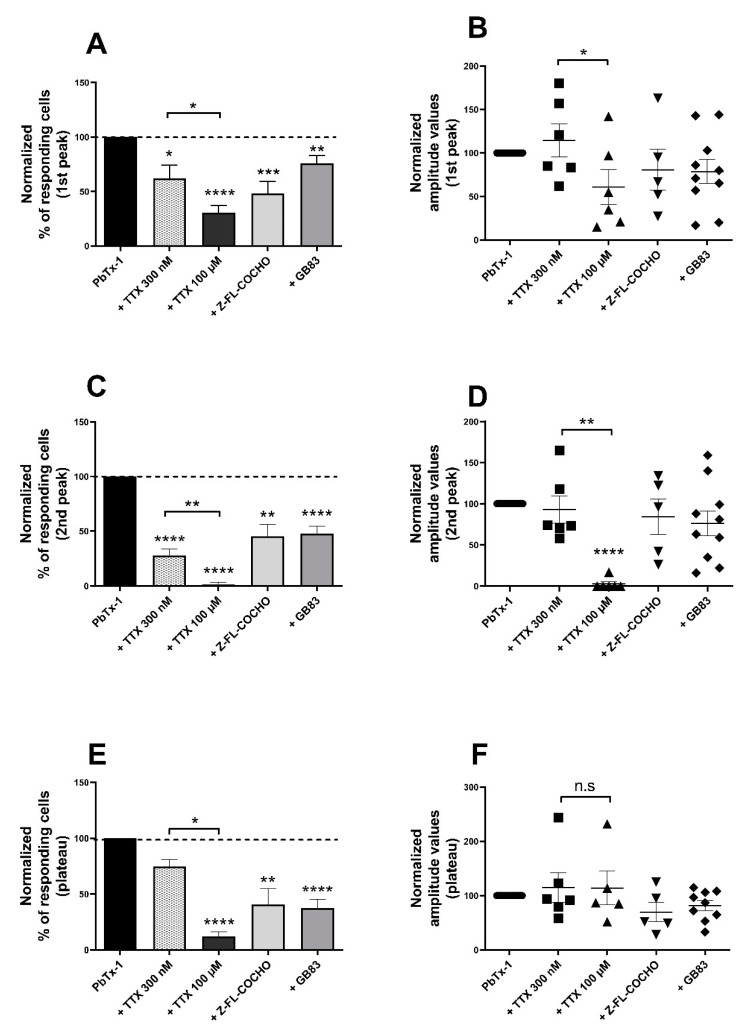
Na_v_ channels (TTX-s and TTX-r), Cat-S and PAR2 mediate PbTx-1-induced [Ca^2+^]_i_ increase in sensory neurons. Normalized percentages of responding cells and normalized average amplitude values were recorded for the first transient (**A**,**B**), the second transient (**C**,**D**), and the plateau (**E**,**F**) in response to 1 µM of PbTx-1 alone (control condition, considered 100%, *n* = 15) or with pre-treatment with 300 nM of TTX (*n* = 6), 100 µM of TTX (*n* = 6), 10 nM of Z-FL-COCHO (*n* = 9) and 5 µM of GB83 (*n* = 9), expressed as mean ± SEM. The “*n*” represents the number of experiments in which 50 to 150 neuronal cells were included in the analysis. In the right panel, each symbol represents the normalized mean of the maximal amplitude values from the responding neurons obtained in each experiment. Statistical analysis was performed with a Kruskal–Wallis followed by Dunn’s test for multiple comparisons between control and each condition, and Mann–Whitney between the condition pre-treatment with TTX 300 nM and the condition pre-treatment with TTX 100 µM. * *p* < 0.05, ** *p* < 0.01, *** *p* < 0.001 and **** *p* < 0.0001. [Ca^2+^]_i_: intracellular calcium concentration; Cat-S: cathepsin S; Na_v_: voltage-gated sodium channel; ns: non-significant; PAR2: protease-activated receptor 2; PbTx-1: brevetoxin-1; TTX: tetrodotoxin; TTX-r: TTX-resistant; TTX-s: TTX-sensitive.

To study whether Na_v_ channels (TTX-s and TTX-r), Cat-S and PAR2 were involved in the neuronal [Ca^2+^]_i_ increase to PbTx1, we used TTX at 300 nM and 100 µM [61], Z-FL-COCHO 10 nM and GB83 5 µM [62], respectively. Effect of each antagonist were assessed separately on the PbTx-1-induced [Ca^2+^]_i_ increase. A total of two parameters were recorded for each phase: (1) the percentage of responding cells to PbTx-1 and (2) the associated amplitude of the response. Both parameters were normalized to those obtained without antagonist pretreatment (i.e., PbTx-1 alone). Figure 2A,C,E represent the normalized percentage of responding cells of the first and second transients and the plateau respectively. Figure 2B,D,F show the associated normalized amplitude values of each phases.

TTX 300 nM (targeting the TTX-s Na_v_) and TTX 100 µM (targeting the TTX-s Na_v_ and the TTX-r Na_v_ channels) significantly decreased the percentage of cells responding to PbTx-1 with the first transient (62.20 ± 12.5%; 30.5 ± 6.4%; of cells responding compared to PbTx-1 alone (100%), respectively). The decrease of percentage of responding cells with the second transient was more pronounced by TTX 100 µM than by TTX 300 nM (28.2 ± 5.4%; 1.7 ± 1.7% of cells responding compared to PbTx-1 alone (100%), respectively). The percentage of responding cells with a plateau was slightly diminished by 300 nM of TTX and strongly diminished by 100 µM of TTX (74.8 ± 6.4%; 12.4 ± 3.9% of cells responding compared to PbTx-1 alone (100%), respectively) (Figure 2A,C,E). In addition, TTX 100 µM strongly decreased the amplitude of the second transient (amplitude reduced to 2.0 ± 2.9% of that of PbTx-1 alone (100%)), whereas 300 nM of TTX did not (Figure 2D). We compared the effects between TTX 300 nM versus TTX 100 µM; a significant difference was recorded for both percentages of responding cells and associated amplitude values of each phase, except for the amplitude values of the plateau suggesting the involvement of TTX-r Na_v_, in addition of TTX-s Na_v_, in [Ca^2+^]_i_ induced by PbTx-1.

We tested the involvement of PAR2 and its agonist Cat-S in order to determine if they were also involved in [Ca^2+^]_i_ induced by PbTx-1. Z-FL-COCHO and GB83 significantly decreased the percentage of cells responding to PbTx-1 with the first transient (48 ± 11.1%; 76 ± 7.4% of responding cells compared to PbTx-1 alone (100%)), with the second transient (45.2 ± 11.1%; 48.9 ± 7.4% of responding cells compared to PbTx-1 alone (100%)) and the plateau (41 ± 13.8%; 38 ± 7.7% of responding cells compared to PbTx-1 alone (100%)) (Figure 2A,C,E, respectively). Z-FL-COCHO and GB83 did not significantly modify the amplitude values of responding cells of each phase (Figure 2B,D,F). Together, these data suggest the critical role of Na_v_ channels including TTX-s and TTX-r Na_v_, Cat-S and PAR2 in the PbTx-1 signaling pathway in sensory neurons.

### 3.3. PbTx-1 Induced Cat-S- and PAR2-Dependent SP Release from Co-Cultured Sensory Neurons and Keratinocytes

**Figure 3:**
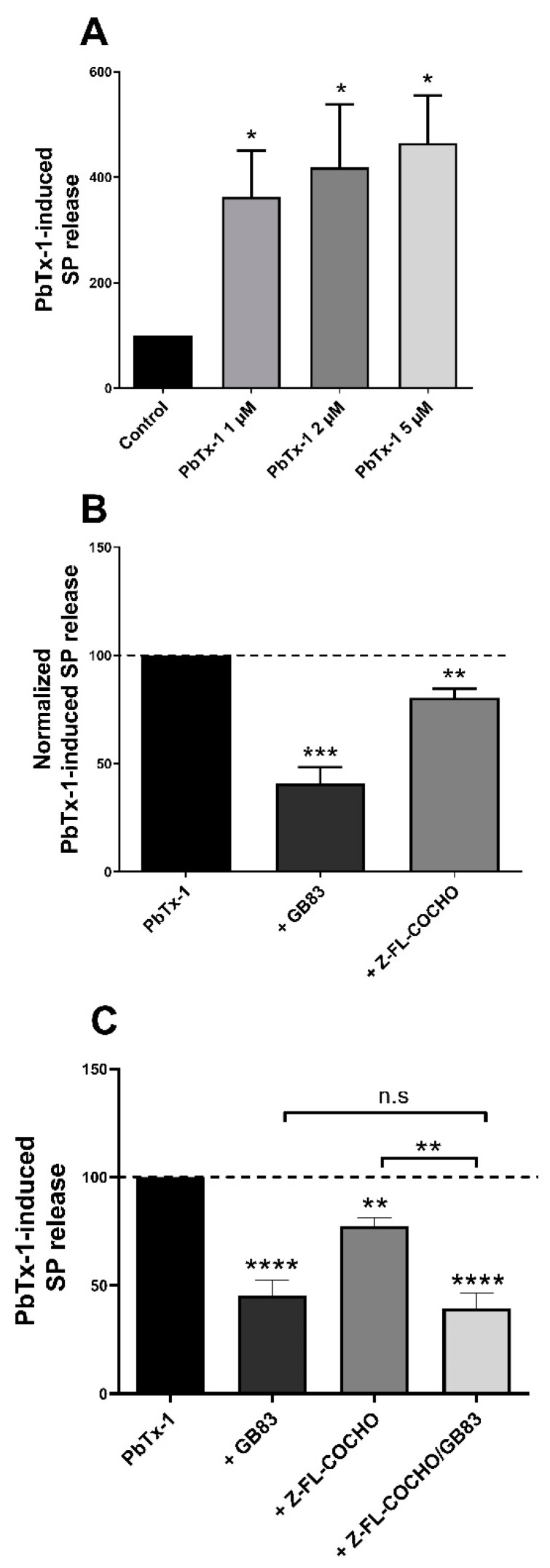
Effects of GB83 and Z-FL-COCHO on PbTx-1-induced SP release from co-cultured sensory neurons and keratinocytes. (**A**) Co-cultures were treated for 90 min with PbTx-1 1 µM (*n* = 4), 2 µM (*n* = 6) and 5 µM (*n* = 5). Data were normalized according to the control condition (100%) and expressed as the mean ± SEM and statistically analyzed with one-tailed paired t-test between PbTx-1 condition versus control. * *p* < 0.05. (**B**) GB83 and Z-FL-COCHO were pre-incubated 10 min before 1 µM PbTx-1 for 90 min. Normalized data were expressed as the mean ± SEM of 7 experiments and analyzed using a one-way ANOVA followed by a Dunnet post-test for multiple comparisons to control. ** *p* < 0.01 and *** *p* < 0.001. (**C**) GB83, Z-FL-COCHO or the combination GB83/Z-FL-COCHO were pre-incubated 10 min before 1 µM PbTx-1 for 90 min. Data were expressed as the mean ± SEM of four experiments and statistically analyzed using an one-way ANOVA followed by a Holm–Sidak test for multiple comparisons to control and between condition (GB83/Z-FL-COCHO was compared to GB83 alone and to Z-FL-COCHO alone). ** *p* < 0.01, *** *p* < 0.001 and **** *p* < 0.0001. ns: non-significant; PbTx-1: brevetoxin-1; SP: substance P.

Following 90 min of treatment with increasing concentrations of PbTx-1, we assessed the ability of PbTx-1 to induce SP release (Figure 3A). No decrease of cell viability after PbTx-1 treatment at 1 µM (99 ± 3.4% compared to control condition (medium, considered as 100%)) and 2 µM (104 ± 1.7% compared to control condition (100%)) was elicited while 5 µM induced a slight decrease (94 ± 2.1% compared to control condition (100%)) of cell viability. PbTx-1 induced a significant increase in SP release in a concentration-dependent manner (363 ± 87.3% at 1 µM; 418 ± 119% at 2 µM and 465 ± 90% at 5 µM). Thus, our results show that PbTx-1 induced up to 4-fold increase of SP release from a co-culture model of sensory neurons and human keratinocytes.

To assess the involvement of PAR2 and Cat-S in the PbTx-1-induced SP release from the co-culture model, we tested the effects of the specific antagonists GB83 and Z-FL-COCHO respectively. Both antagonists significantly reduced the PbTx-1-induced SP release to 41 ± 7.4% and to 80 ± 4.1% (Figure 3B).

In order to know whether Cat-S was responsible for PAR2 activation in PbTx-1-induced SP release, we compared the inhibitory effect of combined GB83/Z-FL-COCHO to GB83 alone. The inhibitory effect of GB83/Z-FL-COCHO (40 ± 6.9%) did not significantly differ from that of GB83 alone (45 ± 6.9%) (Figure 3C). These data indicate that Cat-S and PAR2 belonged to the same pathway. In contrast, the inhibitory effect of GB83 alone (40 ± 6.8%) was significantly higher than that of Z-FL-COCHO alone (78 ± 3.7%) suggesting that Cat-S was not the only mediator of PbTx-1-induced PAR2 activation.

### 3.4. P-CTX-2 Induced PAR2 Internalization Independtly from Cat-S in Sensory Neurons

**Figure 4:**
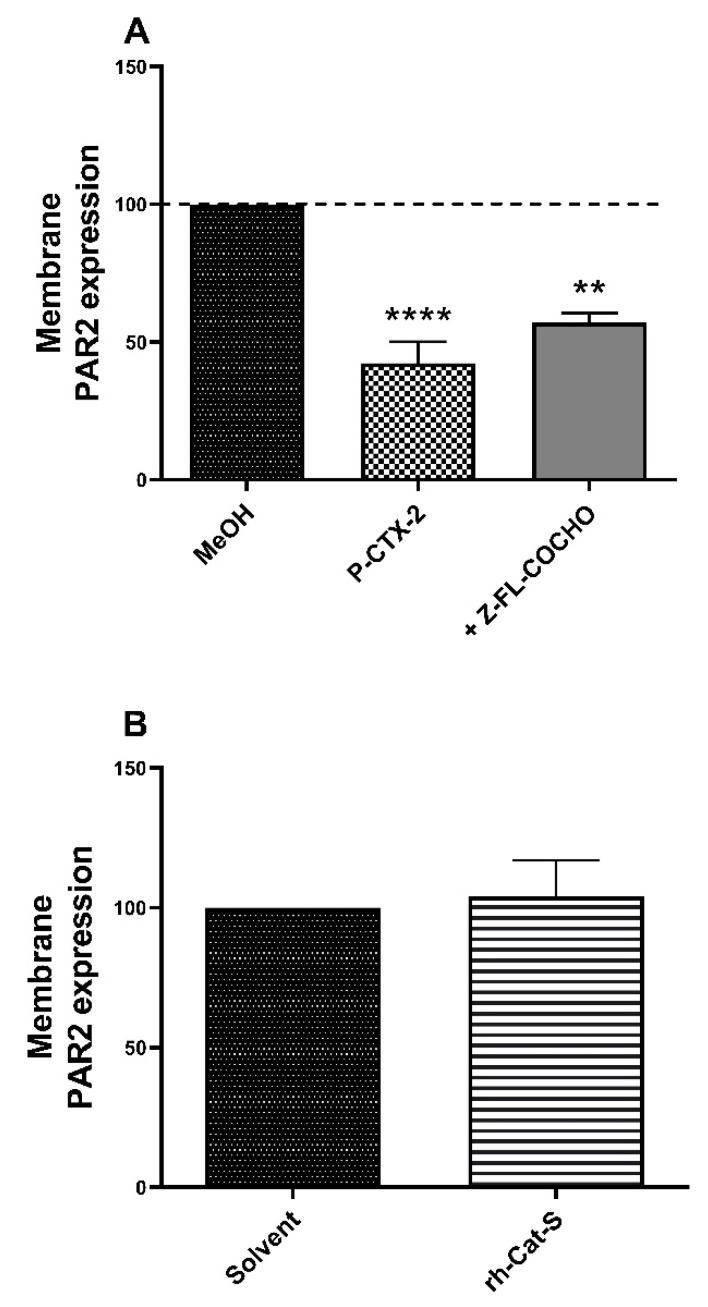
PAR2 internalization induced by P-CTX-2 is Cat-S-independent. (**A**) Effects of 10 nM of P-CTX-2 with or without pre-treatment of 10 nM of Z-FL-COCHO on PAR2 membrane expression in DRG neurons. Normalized data were expressed as mean ± SEM of 3 experiments and statistically analyzed using a one-way ANOVA followed by a Holm-Sidak test for multiple comparisons to control and between P-CTX-2 and P-CTX-2/Z-FL-COCHO. ** *p* < 0.01, **** *p* < 0.0001. (**B**) Effects of human recombinant Cat-S on the PAR2 membrane expression in differentiated human keratinocytes. Five hundred nM of human recombinant Cat-S were added for 20 min. Normalized data were expressed as mean ± SEM of two experiments. Cat-S: cathepsin S; CTX: ciguatoxin; P-CTX-2: Pacific-CTX-2; PAR2: protease-activated receptor 2; rh-Cat-S: human recombinant cathepsin S.

To asses PAR2 membrane expression, non-permabilized DRG neurons were used and exposed to P-CTX-2 with or without Z-FL-COCHO to study Cat-S involvement (Figure 4A). P-CTX-2 induced a significant decrease in the membrane expression of PAR2 (42.3 ± 7.9% compared to control). Pretreatment with Z-FL-COCHO 10 nM did not prevent the decrease in the PAR2 membrane expression (43% ± 3.6%). In order to confirm in primary culture that Cat-S as a biased agonist of PAR2 is unable to internalize PAR2, we treated human keratinocytes with 500 nM of human recombinant Cat-S, which did not modify the PAR2 membrane expression (Figure 4B). These results indicate that P-CTX-2 induced PAR2 internalization and suggest no role of Cat-S in this effect. Thus, these results suggest that Cat-S was not the only mediator of the PAR2-dependent sensory effects induced by P-CTX-2, as by PbTx-1.

### 3.5. PKC, PKA, IP3R and TRPV4 Are Involved in the [Ca^2+^]_i_ Increase Induced by PbTx-1 in Sensory Neurons

**Figure 5:**
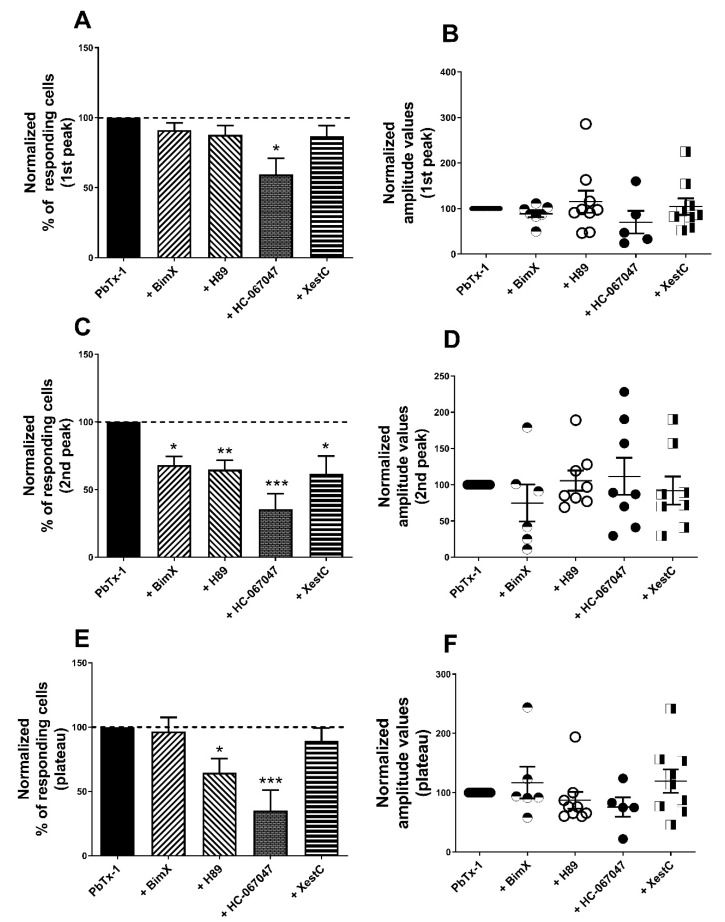
Effects of PKC, PKA, IP3R and TRPV4 antagonists on PbTx-1-induced [Ca^2+^]_i_ increase in sensory neurons. Normalized percentages of responding cells and normalized associated amplitude values recorded for the first transient (**A**,**B**), the second transient (**C**,**D**) and the plateau (**E**,**F**) in response to 1 µM of PbTx-1 alone (control condition (100%), *n* = 15) or with pretreatment with 10 µM of BimX (*n* = 7), 5 µM of H89 (*n* = 9), 10 µM of HC-067047 (*n* = 5), and 5 µM of XestC (*n* = 9) were expressed as mean ± SEM. The “*n*” represents the number of experiments in which 50 to 150 neuronal cells were included in the analysis. In the right panels, each symbol represents the normalized mean of the maximal amplitude values from the responding neurons obtained in each experiment. The statistical analysis was performed with performed with a Kruskal–Wallis followed by Dunn’s test for multiple comparisons between control and each condition. * *p* < 0.05, ** *p* < 0.01 and *** *p* < 0.001. BimX: bisindolylmaleimide X hydrochloride; IP3R: inositol triphosphate receptor; PbTx-1: brevetoxin-1; PKA: protein kinase A; PKC: protein kinase C; TRPV4: transient receptor potential vanilloid 4; XestC: xestospongin C.

To decipher the downstream signaling pathway of PAR2 induced by PbTx-1, we studied the involvement of PKC, PKA and IP3R in the PbTx-1-induced [Ca^2+^]_i_ increase in sensory neurons. We performed single cell cytosolic calcium video imaging experiments using specific antagonists: bisindolylmaleimide X hydrochloride (BimX) for PKC [63], H89 for PKA and xestospongin C (XestC) for IP3R. None of the antagonists decreased the percentage of cells responding to PbTx-1 with the first transient (Figure 5A) however all significantly reduced the percentage of cells responding to PbTx-1 with the second transient (to 68 ± 6.2%; 65 ± 6.8%; 62 ± 13%, compared to PbTx-1 alone (100%)) (Figure 5C). In addition, H89 significantly decreased the percentage of responding cells with the plateau (65 ± 6.8% compared to PbTx-1 alone, (100%)) (Figure 5E). None of the antagonists significantly changed the amplitude values of the three phases of the calcium response (Figure 5B,D,F). Inhibition of PKC, PKA and IP3R prevent the [Ca^2+^]_i_ increase induced by PbTx-1 in several sensory neurons (i.e., decrease the percentage of responding cells to PbTx-1) but did not modify the amplitude of the response in other neuronal cells. Taken together, these results suggest the involvement of PKC and IP3R in the second transient of PbTx-1-induced [Ca^2+^]_i_ increase in sensory neurons, while PKA was involved both in the second transient and the plateau.

To study the involvement of TRPV4 in the PbTx-1-induced [Ca^2+^]_i_ increase in sensory neurons, we used HC-067047 [63]. HC-067047 significantly reduced the PbTx-1-induced [Ca^2+^]_i_ increase in the first transient (to 59 ± 12%), the second transient (to 36 ± 11.5%) and the plateau (to 35 ± 16%) (Figure 5A,C,E). No significant modification of amplitude values was recorded, regardless of the phase of the calcium signal (Figure 5B,D,F). These results suggest the involvement of TRPV4 in the [Ca*^2^*^+^]_i_ increase induced by PbTx-1.

### 3.6. PKC, PKA, IP3R, and TRPV4 Are Involved in the SP Release Induced by PbTx-1 in the Co-Culture

**Figure 6:**
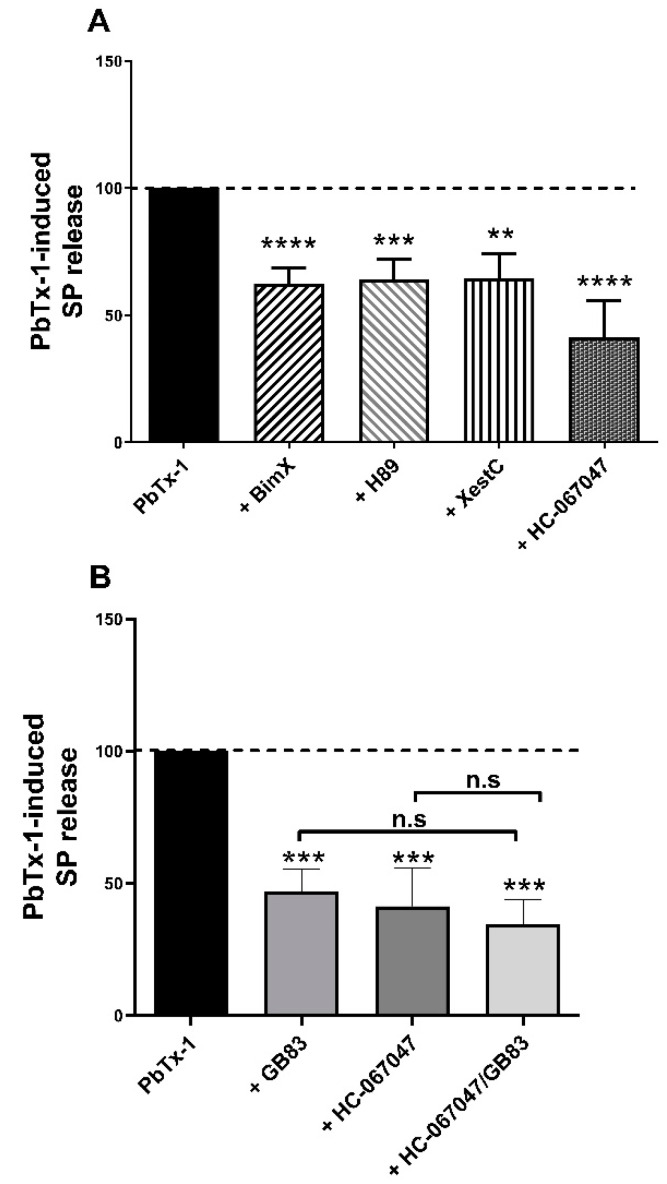
PbTx-1-induced SP release is mediated by PAR2 and TRPV4 in co-culture. (**A**) Normalized levels of SP from supernatants of co-culture in response to 1 µM of PbTx-1 alone (control condition, *n* = 7) or with pre-treatment with 10 µM of BimX (*n* = 5), with 5 µM of H89 (*n* = 5), with 10 µM of HC-067047 (*n* = 3), and with 5 µM of XestC (*n* = 4) were expressed as mean ± SEM. The data were statistically analyzed using a one-way ANOVA for multiple comparisons to control followed by a Dunnet post-test. ** *p* < 0.01, *** *p* < 0.001, **** *p* < 0.0001. (**B**) Normalized levels of SP in supernatants of co-culture in response to PbTx-1 1 µM alone (control condition, *n* = 3) or with pre-treatment with 5 µM of GB83 (*n* = 3), with 10 µM of HC-067047 (*n* = 3), and with GB83/HC-067047 (*n* = 3) were expressed as mean ± SEM. The data were analyzed using a one-way ANOVA followed by a Holm–Sidak test for multiple comparisons (GB83/HC-067047 compared to GB83 alone and HC-067047 alone). *** *p* < 0.001. BimX: bisindolylmaleimide X hydrochloride; IP3R: inositol triphosphate receptor; ns: non-significant; PAR2: protease-activated receptor 2; PKA: protein kinase A; PKC: protein kinase C; PbTx-1: brevetoxin-1; SP: substance P; TRPV4: transient receptor potential vanilloid 4; XestC: xestospongin C.

Based on our calcium experiment results, we explored the effects of pretreatments with PKC, PKA, IP3R and TRPV4 antagonists in the PbTx-1-induced SP release from the co-culture supernatants. PbTx-1-induced SP release was significantly prevented by BimX (reduced to 37 ± 5.9%), H89 (reduced to 36 ± 8.1%), XestC (reduced to 36 ± 9.7%) and HC-067047 (reduced to 59 ±14.5%) (Figure 6A). These findings suggest that PKC, PKA, IP3R and TRPV4 are involved in the SP release induced by PbTx-1 from the co-culture model.

We further assessed whether TRPV4 and PAR2 belonged to the same pathway in the PbTx-1-induced SP release. Pretreatment with a combination of GB83 and HC-067047 did not induce additional decrease in PbTx-1-induced SP release (reduced to 35 ± 9.2%) in conditions pre-treated with GB83 alone (reduced to 47 ± 8.2%) and HC-067047 alone (reduced to 41 ± 14.5%) (Figure 6B). This result indicates that PAR2 and TRPV4 belonged to the same signaling pathway in the PbTx-1 induced SP release and thus suggests that TRPV4 may be sensitized in a PAR2-dependent manner by PbTx-1.

## 4. Discussion

PbTxs induce sensory disturbances [1,2]. The objectives of this study were to (i) assess whether PbTx-1 induces [Ca^2+^]_i_ increase in sensory neurons and SP release from the co-culture model; (ii) study the involvement of Na_v_, PAR2 and Cat-S in those effects; (iii) identify signaling actors involved downstream of PAR2 activation induced by PbTx-1 in sensory neurons. We were able to demonstrate that, similarly to P-CTX-2, Cat-S and PAR2 are key actors in PbTx-1-induced [Ca^2+^]_i_ increase and SP release. In addition, we demonstrated the involvement of PKC, PKA, IP3R and TRPV4 downstream of PAR2 activation induced by PbTx-1 in DRG neurons.

### 4.1. PbTx-1 Induce [Ca^2+^]_i_ Increase and SP Release from Sensory Neurons and Co-Culture Model

Different profiles of PbTx-1-induced [Ca^2+^]_i_ increase in sensory neurons indicate that different subpopulations of neurons were present in our culture. The PbTx-1-induced calcium response in sensory neurons showed a three phases corresponding to the three phases observed in P-CTX-2-induced calcium response in sensory neurons [29]. Almost 54% of rat sensory neurons responded to PbTx-1 matching the proportions shown in mouse DRG neurons responding P-CTX-1B at 1 nM [64], and in rat DRG neurons responding to 10 nM of P-CTX-2 [29]. Notably, 1 µM of PbTx-1 is a relevant concentration, given that PbTx-1 is about 500 times less potent than the P-CTX-1B and 50 times less potent than P-CTX-2 [21,65].

In the present model, PbTx-1 at µM concentrations was able to induce a concentration dependent SP release (Figure 3A). It has been shown that CTXs at nM concentrations induced SP release from several sensory models including murine skin and DRG neurons [25,66,67]. Furthermore, PbTx-3 is able to induce CGRP release from motor fibers [68]. Neuropeptides including SP can be released by sensory neurons in the periphery and/or in the dorsal horn of the spinal cord [26,69] where they transmit non histaminergic pruriceptive and nociceptive information [70]. Our results suggest that SP is a key mediator of PbTx-1-induced sensory symptoms.

### 4.2. Mechanisms Involved in the [Ca^2+^]_i_ Increase and SP Release Induced by PbTx-1

Sensory disturbances (e.g., paresthesia and cold dysesthesia) are likely attributable to Na_v_ activation by PbTxs and CTXs [64,71,72,73]. A total of 300 nM and 100 µM TTX significantly decreased the PbTx-1 [Ca^2+^]_i_ increase in DRG neurons in the first transient suggesting the role of TTX-s and TTX-r Na_v_ in the initiation of PbTx-1-induced [Ca^2+^]_i_ increase. Our results highlight the significantly higher involvement of TTX-r Na_v_ compared to TTX-s Na_v_ in the three phases of the calcium response induced by PbTx-1. TTX-r Na_v_, including Na_v_ 1.8 and Na_v_ 1.9, particularly expressed in the peripheral nervous system [74], mediate itch and pain [75]. Our results are consistent with the role of TTX-r Na_v_ observed with the P-CTX-2 [25], and the preferential activation of Na_v_ 1.8 [64,76] and Na_v_ 1.9 [66]. Our results suggest a critical role of TTX-r Na_v_ and the additional role of TTX-r Na_v_ in the [Ca^2+^]_i_ increase induced by a PbTx in sensory neurons. Wide expression of TTX-r Na_v_, particularly in the rat DRG neurons between 2 and 5 postnatal days [77], and the additional role of TTX-r Na_v_ in the [Ca^2+^]_i_ increase induced by PbTx-1 highlight those receptors as a potential target to treat sensory disorders.

Pretreatment with GB83 inhibited PbTx-1-induced [Ca^2+^]_i_ increase in sensory neurons and SP release from co-culture of sensory neurons and keratinocytes, indicating the involvement of PAR2. PAR2 is expressed by DRG sensory neurons releasing SP and CGRP [26] and by keratinocytes [30,78]. In a traumatic or inflammatory context, several proteases are released by primary afferent nerve fibers or neighboring cells. They activate PAR2, which in turn induces a [Ca^2+^]_i_ increase and subsequently activates the neuropeptide release that causes neurogenic inflammation [26]. PAR2 can also sensitize TRP channels (TRPV1 [42,45,46], TRPA1 [47] and TRPV4 [48]) contributing to release neuropeptides and induces pain/pruritus. Blocking or silencing PAR2 attenuates or prevents hyperalgesia [79,80]. Since PAR2 is a central actor of PbTx-1 induced [Ca^2+^]_i_ increase and SP release, our results highlight PAR2 as a potential target to treat sensory disorders induced by PbTxs.

As PbTxs are unlikely to exert direct protease activity, we hypothesized that PAR2 activation is indirectly induced by proteases, especially Cat-S in a Na_v_-dependent manner [29,81]. Cat-S activates PAR2 in vitro [34] and induces in vivo itch, pain and inflammation in a PAR2-dependent manner [53,56,82]. Moreover, Cat-S plays a major role in the microglial activation and maintenance of neuropathic and chronic pain [83,84]. At a basal level, microglial cells and macrophages are the main source of Cat-S [85]. After peripheral nerve injury, Cat-S is up-regulated in DRG neurons and Schwann cells [86] as well as in keratinocytes after stimulation [87]. In addition, Cat-S and PAR2 are involved in the [Ca^2+^]_i_ increase and SP release induced by P-CTX-2 (i.e an analog of PbTx-1) in sensory neurons [29]. Pretreatment with Z-FL-COCHO (a Cat-S inhibitor) blocks PbTx-1-induced [Ca^2+^]_i_ increase in sensory neurons and SP release from a co-culture of sensory neurons and keratinocytes. Moreover, Cat-S activity is increased in the co-culture after treatment with P-CTX-2 and PbTx-1 [29]. Together, those data suggest that the Cat-S is involved in PbTx- signaling in DRG sensory neurons. Since Cat-S is a critical mediator involved in sensory disorders, its involvement in PbTx-1-induced SP release suggests a contribution to sensory disorders. Blocking Cat-S could represent a new therapeutic target to relieve sensory symptoms induced by PbTx-1. Another brevetoxin analog, namely, PbTx-2, has been previously identified as a ligand and as an inhibitor of the cysteine proteases cathepsin L and papain [88]. In contrast, the present study and our previous work [29] demonstrate that P-CTX-2 and PbTx-1 increase Cat-S activity. This discrepancy could stem from the use of different toxin analogs (PbTx-2 vs. PbTx-1) and proteases (cathepsin L vs. Cat-S) or the employed methods (computational method and a protease activity assay in a cell-free model vs. direct measurement of Cat-S activity in co-culture supernatants).

### 4.3. Internalization of PAR2 Induced by P-CTX-2 Is Not Mediated by Cat-S in Sensory Neurons

At a basal level, PAR2 is mainly expressed at the cell membrane [49]. Next to canonical activation, PAR2 is internalized within a few minutes [49]. Internalization of PAR2 belongs to the canonical pathway but not the biased pathway [53]. In a previous publication using confocal microscopy and immunocytochemistry, we showed that P-CTX-2 and PbTx-1 induced a Na_v_-dependent partial PAR2 internalization in primary keratinocytes and DRG neurons [29]. In the present study, we showed that [Ca^2+^]_i_ increase and SP release evoked by PbTx-1 involved the biased PAR2 agonist Cat-S. Previously Zhao et al. [53] showed that Cat-S did not internalize PAR2 in a PAR2 transfected cell line. Here, we confirmed that human recombinant Cat-S is not able to induce PAR2 internalization in differentiated primary human keratinocytes. Our results showed that pretreatment with Z-FL-COCHO did not prevent P-CTX-2 induced PAR2 internalization in sensory neurons. Thus, PAR2 internalization occurs independently from Cat-S. This suggests the involvement of other mediators, which remain to be identified, in P-CTX-2-induced PAR2 activation. Together, these results strongly suggest the implication of both biased (via Cat-S) and classical (evidenced by internalization) PAR2 pathways triggered by PbTx-1 and P-CTX-2 in sensory neurons.

Our results imply that, in addition to Cat-S, other mediators are involved in PbTx-1-induced PAR2 activation. Kallikrein-related peptidases (KLK) are a family with, so far, 15 known members. They are expressed by the epidermal cells and appendages [89,90,91]. They are implicated in keratinocyte proliferation and differentiation [92] and induce itch [93,94]. Among human serine proteases, KLK5, KLK6 and KLK14 induce PAR2 signaling in human models in vitro but not KLK7 and KLK8 [91,95]. In contrast, in rat-derived KNRK transfected cells, KLK8 is not able to induce calcium signaling. This difference may be related to different methods or minor differences between species. KLK5, KLK6 and KLK14 were able to induce [Ca^2+^]_i_, increase comparable to trypsin [95,96]. Moreover, KLK14 induces PAR2 internalization and interaction between β-arrestin and PAR2 [96] indicating downstream canonical pathway involvement, comparable to trypsin [38]. Further experiments are necessary to determine the contribution of several KLKs to PAR2 activation and/or internalization in sensory neurons after PbTx-1 treatment, especially KLK5, KLK6 and KLK14

### 4.4. PKC, PKA, IP3R and TRPV4 Involvement in PbTx-1-Induced [Ca^2+^]_i_ Increase and SP Release

Next we studied the role of a number of downstream actors in the canonical and biased PAR2 activation; PKC and IP3R belonging to the canonical pathway [41,42,48]; PKA [42,48,97] and TRPV4 [48,53] which are activated in both PAR2 biased and canonical pathways. PAR2 is co-expressed in DRG neurons with PKC [47], PKA [42] and TRPV4 [48]. Our results showed that both actors are implicated in PbTx-1-induced [Ca^2+^]_i_ increase in sensory neurons. Some antagonists prevent the [Ca^2+^]_i_ increase induced by PbTx-1 in several sensory neurons but did not modify the amplitude of the response in other neuronal cells highlighting different subpopulations. This fact suggests the involvement of PKA, PKC and IP3R in subpopulation(s) of sensory neurons

**Scheme 1:**
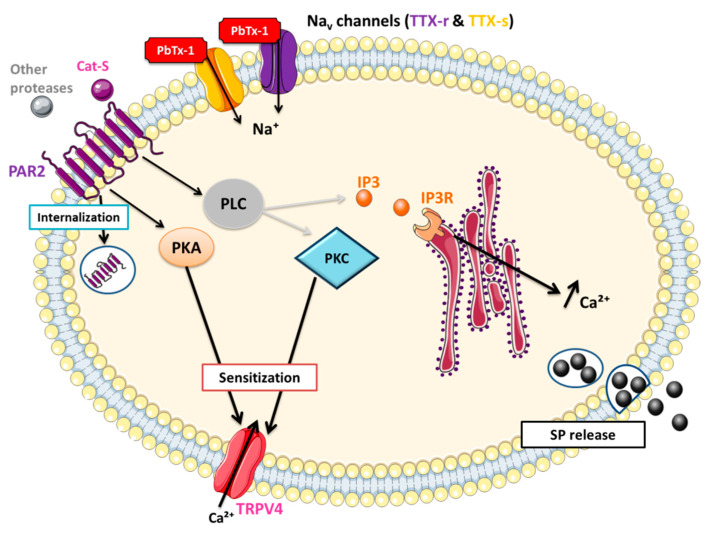
Schematic representation of PbTx-1-induced signaling in sensory neurons. PbTx-1 binds Na_v_ channels (TTX-s and TTx-r) and increases Cat-S activity (mechanisms not fully understood), which activates PAR2 and its biased pathway including PKA and TRPV4. Other mediators activate PAR2 internalization, PKC and IP3R. PAR2 activation leads to TRPV4 sensitization through PKA and/or PKC. IP3R and TRPV4 mediate [Ca^2+^]_i_ increase, which in turn leads to SP release. Grey arrow: not demonstrated here. Ca^2+^: calcium ion; Na^+^: sodium ion; Cat-S: cathepsin S; IP3: inositol triphosphate; IP3R: inositol triphosphate receptor; Na_v_: voltage-gated sodium channel; PAR2: protease-activated receptor 2; PbTx-1: brevetoxin-1; PKA: protein kinase A; PKC: protein kinase C; PLC: phospholipase C; SP: substance P; TRPV4: transient receptor potential vanilloid 4; TTX: tetrodotoxin; TTX-r: TTX-resistant; TTX-s: TTX-sensitive.

Results of SP release experiments conducted with BimX, H89, XestC and HC-067047 suggested the involvement of PKC, PKA, IP3R and TRPV4 in the SP release induced by PbTx-1 in our co-culture model. These results confirm the involvement of at least the canonical PAR2 signaling pathway following PAR2 activation by PbTx-1. In addition, we identified TRPV4 as a receptor sensitized by PbTx-1 in sensory neurons, suggesting that it could contribute to PbTx-1-induced sensory disturbances. The results highlighted in this study are summarized in Scheme 1.

Interestingly, IP3R were shown to be activated in myotubes of rats treated with PbTx-3 and P-CTX-1B [98,99]. Our results showed their involvement in the PbTx-1-induced [Ca^2+^]_i_ increase and SP release. Since we did not assess whether IP3R and PAR2 belonged to the same pathway, we cannot exclude that PAR2-independent (e.g., voltage-gated calcium channels (Ca_v_)-dependent) mechanisms leading to IP3R activation. Further experiments are necessary to assess PAR2 dependency of IP3R involvement after PbTx-1 treatment from sensory neurons and our co-culture model.

TRPV4 is a non-selective and permeable Ca^2+^ channel regulated by diverse array of stimuli such as temperature, phorbol esters (e.g., 4α-phorbol 12,13-didecanoate), arachidonic acid, hypoosmotic swelling [100]. Calcium influx through TPRV4 can activate Ca^2+^-dependent PLC isoforms, and induce Ca^2+^ release from stores by activating IP3R or ryanodine receptors (RyR) amplifying the Ca^2+^ signal. Ca^2+^ influx through TRPV4 may contribute to the [Ca^2+^]_i_ increase and the SP release induced by PbTx-1. A bidirectional interaction between TRPV4 channels and IP3Rs [101] or RyR [102] is established and TRPV4 plays a central role in Ca^2+^ signaling [103]. Here we focused on the sensitization of TRPV4 after PAR2 activation [53].

Inhalation of PbTx aerosols induces respiratory symptoms especially in persons with underlying airway diseases such as asthma. Upper-airway symptoms include cough, sneezing, rhinorrhea, burning sensation in the nose and throat, associated with facial itch of the skin, watery eyes and neurological symptoms such as headache [9], which support that aerosolized PbTxs exert airway sensory effects. Lower-airway symptoms include chest tightness, wheezing and shortness of breath, reflecting difficulty breathing [5,6,7,8]. Interestingly, in airways, PAR2 activation leads to SP release by nerve endings, which in turn acts directly on smooth muscles to induce bronchoconstriction [104,105,106]. SP expression in nerve fibers is enhanced in asthmatic patients [107]. Thus, our results showing that PbTx-1 induced a Cat-S and PAR2-dependent SP release suggest that this pathway could contribute to respiratory symptoms induced in humans after exposure to aerosolized PbTxs during red tides and be targeted to relieve these symptoms, in addition to sensory disorders.

## 5. Conclusions

Our results showed that PbTx-1 increased [Ca^2+^]_i_ in DRG neurons and SP release from coculture of DRG neurons and keratinocytes. These effects involved Cat-S and PAR2. Cat-S was not the only mediator of PAR2 activation. We decipher the signaling pathway induced by PbTx-1, which involved PKA and TRPV4 that belong, but not exclusively, to the PAR2 biased signaling induced by Cat-S. TRPV4 activation was PAR2-dependent. We also showed that PKC and IP3R, belonging to the PAR2 canonical signaling, are involved in the PbTx-1 increased [Ca^2+^]_i_ and SP release. In the context of human exposure to PbTxs through both oral and inhaled route, our findings suggest that Cat-S and even more PAR2 and TRPV4 might be interesting therapeutic targets to relieve sensory disturbances.

## Supplementary Materials

The following are available online at https://www.mdpi.com/2073-4409/9/12/2704/s1, Table S1: Antagonists—their respective targets and the referenced concentrations.

## Abbreviations

Cat-S	Cathepsin S
CTXs	Ciguatoxins
Na_v_	Voltage-dependent sodium channel
NSP	Neurotoxic shellfish poisoning
PAR2	Protease-activated receptor 2
PbTxs	Brevetoxins
P-CTX-2	Pacific-ciguatoxin-2
PKA	Protein kinase A
PKC	Protein kinase C
SP	Substance P
TRPV4	The transient receptor potential vanilloid 4
TTX	Tetrodotoxin
TTX-r Na_v_	Tetrodotoxin-resistant Na_v_ channel
TTX-s Na_v_	Tetrodotoxin-sensitive Na_v_ channel
