# Supplementary material for: Calcium Increase and Substance P Release Induced by the Neurotoxin Brevetoxin-1 in Sensory Neurons: Involvement of PAR2 Activation through Both Cathepsin S and Canonical Signaling
Source: Cells. 2020 Dec 17;9(12):2704. doi: 10.3390/cells9122704 (PMC7767211; doi:10.3390/cells9122704)
Supplement: Supplementary file 1 [file cells-09-02704-s001.pdf]

cells MDPI

Supplementary Materials:

Reagents: PbTx-1 and tetrodotoxin (TTX) were purchased from Latoxan (Valence, France). A total of two concentrations of TTX were used: 100 µM, which antagonizes both TTX-resistant (TTX-r) and TTX-sensitive (TTX-s) Na_v channels, and 300 nM, which inhibits only TTX-s Na_v channels [25,61,77]. The difference in the inhibitory effects between 100 µM and 300 nM represents the contributory part of TTX-r Na_v-dependent mechanisms. Pure P-CTX-2 was isolated from moray eel (*Gymnothorax javanicus*) livers as previously described [65] and provided by Richard J. Lewis (Institute of Molecular Biosciences, Brisbane, Australia). Stock solution of 1.15 mM of PbTx-1 was prepared in pure methanol (MeOH), aliquoted and dried, and then resolubilized in the medium before use. P-CTX-2 was dissolved in MeOH: water (1:1) to prepare a 10 mM stock solution. Culture media were supplemented with 100 mg/mL Normocin® (InvivoGen, Toulouse, France) obtained from Life Technologies (Saint Aubin, France). Keratinocyte-Serum Free Medium (KSFM) was supplemented with L-glutamine, epidermal growth factor (EGF), and bovine pituitary extract (BPE) (Life Technologies, Saint Aubin, France) to obtain a so-called complete KSFM. GB83 was purchased from Axon Medchem (Groningen, Netherlands) and Z-FL-COCHO was purchased from Merck-Milipore (Burlington, USA). Antagonists such as bisindolylmaleimide X hydrochloride (BimX), H89, and HC-067047 were provided by Sigma-Aldrich (Saint Quentin Fallavier, France). Xestospongin C (XestC) was purchased from Abcam (Cambridge, UK) and the human recombinant Cat-S was obtained from R&D systems (Minneapolis, USA). All antagonists and final concentrations used are summarized in Table S1.

Table S1. Antagonists—their respective targets and the referenced concentrations.

Antagonists	Target	Concentration	References
TTX	All Na_v channels	100 μM	[77]
	TTX-sensitive Na_v channels (TTX-s)	300 nM	
GB83	PAR2	5 μM	[62]
HC-067047	TRPV4	10 μM	[63]
Z-FL-COCHO	Cat-S	10 nM	[81]
BimX	PKC	10 μM	[63]
H89	PKA	3 μM	[48]
XestC	IP3R	5 μM	[44,103]
